# Retraction Note: Receptor tyrosine kinase inhibitor Sunitinib and integrin antagonist peptide HM-3 show similar lipid raft dependent biphasic regulation of tumor angiogenesis and metastasis

**DOI:** 10.1186/s13046-020-1538-8

**Published:** 2020-02-17

**Authors:** Jialiang Hu, Wenjing Wang, Chen Liu, Mengwei Li, Edouard Nice, Hanmei Xu

**Affiliations:** 1 State Key Laboratory of Natural Medicines, Ministry of Education, China Pharmaceutical University, Nanjing, 210009 People’s Republic of China; 2 The Engineering Research Center of Synthetic Polypeptide Drug Discovery and Evaluation of Jiangsu Province, Nanjing, 211198 People’s Republic of China; 3 Department of Biochemistry and Molecular Biology, Monash University, Clayton, VIC 3800 Australia

**Retraction Note: J Exp Clin Cancer Res**

**https://doi.org/10.1186/s13046-019-1324-7**

The authors have retracted this article [1] because the bands shown in Fig. 5 panel D for GTP-RhoA/Control, GTP-RhoA/Sunitinib (2 and 64 nM) and GTP-RhoA/HM-3 (4.5 and 72 uM) are not data generated as part of this study. All authors agree to this retraction.
